# Supplementary material for: The Role of Alpha-Synuclein and Other Parkinson’s Genes in Neurodevelopmental and Neurodegenerative Disorders
Source: Int J Mol Sci. 2020 Aug 10;21(16):5724. doi: 10.3390/ijms21165724 (PMC7460874; doi:10.3390/ijms21165724)
Supplement: Supplementary file 1 [file ijms-21-05724-s001.pdf]

Supplementary Materials

The Role of Alpha-Synuclein and Other Parkinson's Genes in Neurodevelopmental and Neurodegenerative Disorders

C. Alejandra Morato Torres [1], Zinah Wassouf [2,3], Faria Zafar [1], Danuta Sastre [1], Tiago Fleming Outeiro [2,3,4,5] and Birgitt Schüle [1,*]

1 Department Pathology, Stanford University School of Medicine, Stanford, CA 94304, USA; aletor1@stanford.edu (C.A.M.T.); fzafar@stanford.edu (F.Z.); danutasastre@aol.com (D.S.)
2 German Center for Neurodegenerative Diseases, 37075 Göttingen, Germany; Zinah.Wassouf@dzne.de (Z.W.); touteir@gwdg.de (T.F.O.)
3 Department of Experimental Neurodegeneration, Center for Biostructural Imaging of Neurodegeneration, University Medical Center Göttingen, 37075 Göttingen, Germany
4 Max Planck Institute for Experimental Medicine, 37075 Göttingen, Germany
5 Translational and Clinical Research Institute, Faculty of Medical Sciences, Newcastle University, Framlington Place, Newcastle Upon Tyne NE2 4HH, UK
* Correspondence: bschuele@stanford.edu; Tel.: +1-650-721-1767

Table of Contents

Figure S1: Comparative genomic hybridization and optical mapping detect size and orientation of *SNCA* copy number variants

Table S1: Partial exonic PARK2 CNV deletion/duplication coordinates for UCSC genome browser custom tracks

Table S2: 22q11.21 deletions coordinates for UCSC genome browser custom tracks

Table S3: (A) 4q22.1deletions (<10MB) coordinates for UCSC genome browser custom tracks. (B) Small *SNCA* deletions/duplication coordinates for UCSC genome browser custom tracks. (C) *SNCA* duplication/ triplication sizes and coordinates for UCSC genome browser custom tracks.

Table S4. Molecular, morphological, functional, and behavioral phenotypes in alpha-synuclein knockout models

References

Figure S1: Comparative Genomic Hybridization and Optical Mapping Detect Size and Orientation of *SNCA* Copy Number Variants

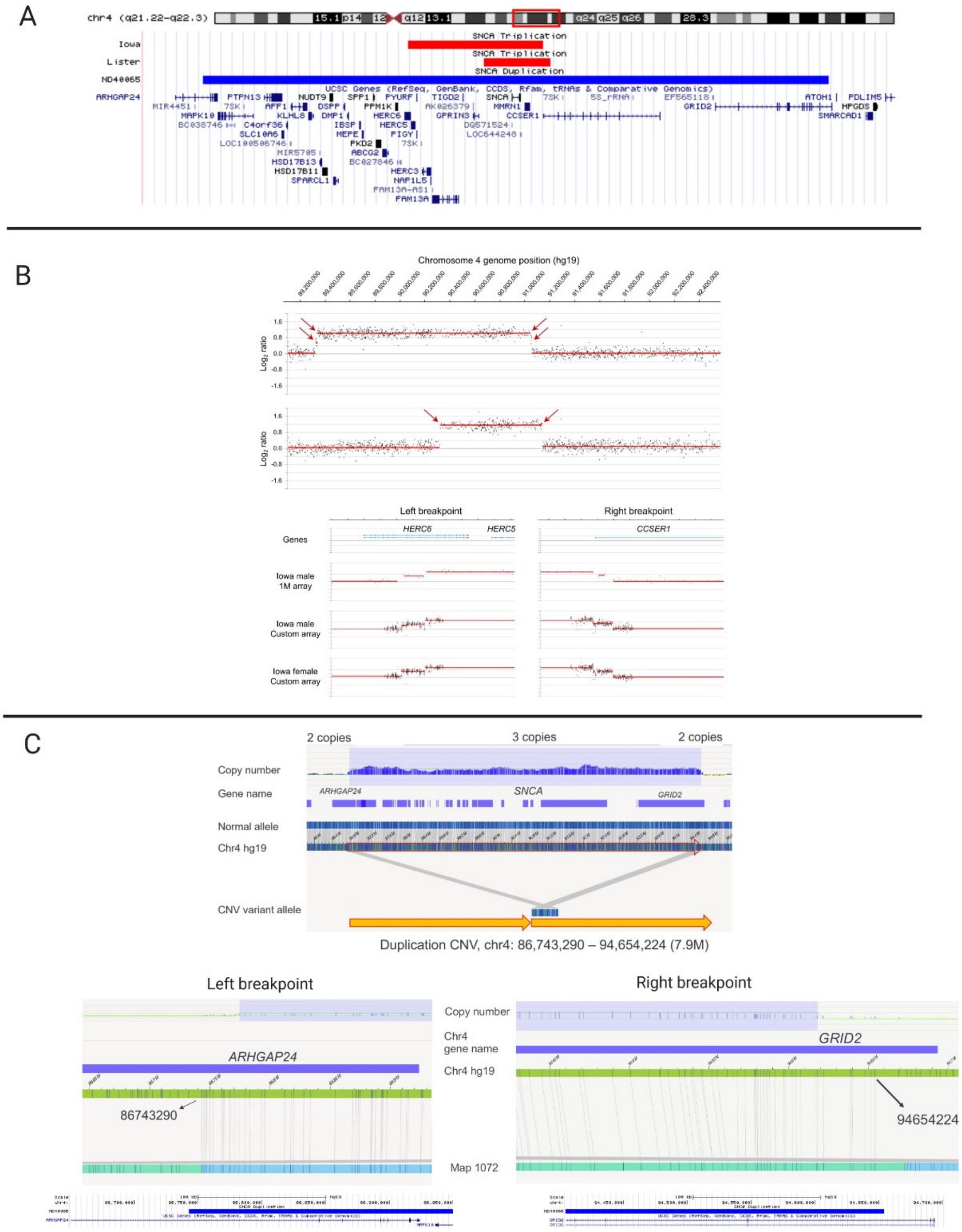

Figure S1: A. UCSC genome browser custom tracks (genome build GRCh37/hg19, February 2009) for two SNCA triplication cases (red) and one SNCA duplication (blue). B. Illustration of comparative genomic hybridization (CGH) for two SNCA triplication cases. The lower panel of B shows the left and right breakpoint with a 'step' that indicated two different recombination events [1]. C. Optical mapping using Saphyr Genome Imaging (Bionano) allows for CNV sizing (resolution 500bp) and orientation of CNVs. Case is from NINDS Cell repository (Cell line ID: ND40065) and presents with a 7.9Mb duplication disrupting the Rho GTPase-activating protein 24 (ARHGAP24) and Glutamate receptor delta-2 (GRID2) gene. (GRID2) is also implicated in autism (https://gene.sfari.org/database/human-gene/GRID2) [2].

Table S1: Partial Exonic PARK2 CNV Deletion/Duplication Coordinates for UCSC Genome Browser Custom Tracks (Genome Build GRCh37/hg19, February 2009)

In this Table S1, we listed genome coordinates for Figure 1. Case data were analyzed from Autism Genome Project (AGP) [3].

Count	Type	Exon	Size (bp)	Chrom.	Start	Stop	ID
1	Deletion	8/9	197,372	chr6	161892760	162090131	6016_3
2	Deletion	5/6	265,769	chr6	162275484	162541252	14103_1780
3	Duplication	3/4	274,643	chr6	162495289	162769931	20163_1672002
4	Duplication	2/3/4	408,545	chr6	162495289	162903833	13100_1173
5	Duplication	2/3/4	389,593	chr6	162511301	162900893	4156_1
6	Duplication	2/3/4	351,673	chr6	162552161	162903833	6362_3
7	Deletion	3/4	226,621	chr6	162580056	162806676	5326_3
8	Deletion	4	41,060	chr6	162588879	162629938	8612_201
9	Deletion	3/4	160,236	chr6	162601734	162761969	2267_1
10	Duplication	3/4	212,453	chr6	162622524	162834976	14220_3530
11	Duplication	3/4	212,453	chr6	162622524	162834976	6347_3
12	Duplication	3	192,238	chr6	162637688	162829925	13119_1364
13	Duplication	3	185,689	chr6	162644237	162829925	5335_3
14	Duplication	2/3	190,740	chr6	162644237	162834976	5431_4
15	Deletion	3	143,617	chr6	162666349	162809965	3382_5

Genome Browser Custom Tracks (GRCh37/hg19)
browser position chr6:161756581-163162564
track name="PARK2 - 6q26" description="PARK2 - 6q26" visibility=2 itemRgb="On"
chr6 161892760 162090131 6016_3 0 . 161892760 162090131 34,139,34
chr6 162275484 162541252 14103_1780 0 . 162275484 162541252 34,139,34
chr6 162495289 162769931 20163_1672002 0 . 162495289 162769931 65,105,225
chr6 162495289 162903833 13100_1173 0 . 162495289 162903833 65,105,225
chr6 162511301 162900893 4156_1 0 . 162511301 162900893 65,105,225
chr6 162552161 162903833 6362_3 0 . 162552161 162903833 65,105,225
chr6 162580056 162806676 5326_3 0 . 162580056 162806676 34,139,34
chr6 162588879 162629938 8612_201 0 . 162588879 162629938 34,139,34
chr6 162601734 162761969 2267_1 0 . 162601734 162761969 34,139,34
chr6 162622524 162834976 14220_3530 0 . 162622524 162834976 65,105,225
chr6 162622524 162834976 6347_3 0 . 162622524 162834976 65,105,225
chr6 162637688 162829925 13119_1364 0 . 162637688 162829925 65,105,225
chr6 162644237 162829925 5335_3 0 . 162644237 162829925 65,105,225
chr6 162644237 162834976 5431_4 0 . 162644237 162834976 65,105,225
chr6 162666349 162809965 3382_5 0 . 162666349 162809965 34,139,34

Table S2: 22q11.21 deletions coordinates for UCSC genome browser custom tracks (genome build NCBI36/hg18, March 2006)

In this Table S2, we listed genome coordinates for Figure 2.

As a reference, we included the classic 22q11.21 deletion region (Velocardiofacial/DiGeorge syndrome) (https://decipher.sanger.ac.uk/syndrome/16#genotype/cnv/21/browser) and the recently characterized critical region for a higher rate of autism (LCR-A to LCR-B) [4].

Case data were analyzed from Autism Genome Project (AGP) [3]. For cases with identical deletions, we grouped cases into groups G1 to G9 (column ID/Group ID).

Count	Type	Size (bp)	Chrom.	Start	Stop	ID/Group ID
1	Deletion	2,578,170	chr22	17241748	19819918	3183_7
2	Deletion	2,537,993	chr22	17257787	19795780	17015_1
3	Deletion	327,829	chr22	17060279	17388108	6246_4
4	Deletion	301,170	chr22	18724527	19025697	4074_1
5	Deletion	294,246	chr22	18719310	19013556	2288_1
6	Deletion	225,582	chr22	17020300	17245882	13112_1293
7	Deletion	216,802	chr22	18794653	19011455	5264_4
8	Deletion	210,493	chr22	18815204	19025697	5382_3
9	Deletion	206,237	chr22	17051550	17257787	
10	Deletion	206,237	chr22	17051550	17257787	
11	Deletion	206,237	chr22	17051550	17257787	G1
12	Deletion	206,237	chr22	17051550	17257787	
13	Deletion	206,237	chr22	17051550	17257787	
14	Deletion	197,508	chr22	17060279	17257787	G2
15	Deletion	197,508	chr22	17060279	17257787	
16	Deletion	194,332	chr22	17051550	17245882	
17	Deletion	194,332	chr22	17051550	17245882	
18	Deletion	194,332	chr22	17051550	17245882	G3
19	Deletion	194,332	chr22	17051550	17245882	
20	Deletion	194,332	chr22	17051550	17245882	
21	Deletion	185,603	chr22	17060279	17245882	
22	Deletion	185,603	chr22	17060279	17245882	
23	Deletion	185,603	chr22	17060279	17245882	
24	Deletion	185,603	chr22	17060279	17245882	G4
25	Deletion	185,603	chr22	17060279	17245882	
26	Deletion	185,603	chr22	17060279	17245882	
27	Deletion	185,603	chr22	17060279	17245882	
28	Deletion	171,395	chr22	17074487	17245882	5264_4
29	Deletion	153,319	chr22	17092563	17245882	5516_3
30	Deletion	150,936	chr22	17051550	17202486	G5
31	Deletion	150,936	chr22	17051550	17202486	
32	Deletion	149,365	chr22	17108422	17257787	G6
33	Deletion	149,365	chr22	17108422	17257787	
34	Deletion	146,360	chr22	17241748	17388108	
35	Deletion	146,360	chr22	17241748	17388108	G7
36	Deletion	146,360	chr22	17241748	17388108	
37	Deletion	142,226	chr22	17245882	17388108	5265_5

38	Deletion	142,207	chr22	17060279	17202486	5388_3
39	Deletion	138,876	chr22	17257787	17396663	13071_843
40	Deletion	137,460	chr22	17108422	17245882	3029_4
41	Deletion	132,045	chr22	17051550	17183595	4220_1
42	Deletion	130,321	chr22	17257787	17388108	
43	Deletion	130,321	chr22	17257787	17388108	
44	Deletion	130,321	chr22	17257787	17388108	G8
45	Deletion	130,321	chr22	17257787	17388108	
46	Deletion	130,321	chr22	17257787	17388108	
47	Deletion	123,316	chr22	17060279	17183595	4222_1
48	Deletion	120,248	chr22	18751164	18871412	5432_3
49	Deletion	110,386	chr22	18569077	18679463	G9
50	Deletion	110,386	chr22	18569077	18679463	
51	Deletion	90,508	chr22	18575591	18666099	4208_1
52	Deletion	46,573	chr22	18317638	18364211	4288_1
53	Deletion	44,554	chr22	18274542	18319096	8703_201
54	Deletion	29,885	chr22	18409878	18439763	20033_1227001
55	Deletion	14,003	chr22	18323020	18337023	1240_3
56	Deletion	10,514	chr22	17742142	17752656	14388_4970
57	Deletion	6,252	chr22	18166460	18172712	14327_4410

Count	Genome Browser Custom Tracks (GRCh37/hg19)
	browser position chr22:16309492-21775472
	track name="22q11.21 deletions" description="22q11.21 deletions" visibility=2 itemRgb="On"
1	chr22 17241748 19819918 3183_7 0 . 17241748 19819918 34,139,34
2	chr22 17257787 19795780 17015_1 0 . 17257787 19795780 34,139,34
3	chr22 17060279 17388108 6246_4 0 . 17060279 17388108 34,139,34
4	chr22 18724527 19025697 4074_1 0 . 18724527 19025697 34,139,34
5	chr22 18719310 19013556 2288_1 0 . 18719310 19013556 34,139,34
6	chr22 17020300 17245882 13112_1293 0 . 17020300 17245882 34,139,34
7	chr22 18794653 19011455 5264_4 0 . 18794653 19011455 34,139,34
8	chr22 18815204 19025697 5382_3 0 . 18815204 19025697 34,139,34
9	chr22 17051550 17257787 G1 0 . 17051550 17257787 34,139,34
10	chr22 17060279 17257787 G2 0 . 17060279 17257787 34,139,34
11	chr22 17051550 17245882 G3 0 . 17051550 17245882 34,139,34
12	chr22 17060279 17245882 G4 0 . 17060279 17245882 34,139,34
13	chr22 17074487 17245882 5264_4 0 . 17074487 17245882 34,139,34
14	chr22 17092563 17245882 5516_3 0 . 17092563 17245882 34,139,34
15	chr22 17051550 17202486 G5 0 . 17051550 17202486 34,139,34
16	chr22 17108422 17257787 G6 0 . 17108422 17257787 34,139,34
17	chr22 17241748 17388108 G7 0 . 17241748 17388108 34,139,34
18	chr22 17245882 17388108 5265_5 0 . 17245882 17388108 34,139,34
19	chr22 17060279 17202486 5388_3 0 . 17060279 17202486 34,139,34
20	chr22 17257787 17396663 13071_843 0 . 17257787 17396663 34,139,34
21	chr22 17108422 17245882 3029_4 0 . 17108422 17245882 34,139,34

22	chr22 17051550 17183595 4220_1 0 . 17051550 17183595 34,139,34
23	chr22 17257787 17388108 G8 0 . 17257787 17388108 34,139,34
24	chr22 17060279 17183595 4222_1 0 . 17060279 17183595 34,139,34
25	chr22 18751164 18871412 5432_3 0 . 18751164 18871412 34,139,34
26	chr22 18569077 18679463 G9 0 . 18569077 18679463 34,139,34
27	chr22 18575591 18666099 4208_1 0 . 18575591 18666099 34,139,34
28	chr22 18317638 18364211 4288_1 0 . 18317638 18364211 34,139,34
29	chr22 18274542 18319096 8703_201 0 . 18274542 18319096 34,139,34
30	chr22 18409878 18439763 20033_1227001 0 . 18409878 18439763 34,139,34
31	chr22 18323020 18337023 1240_3 0 . 18323020 18337023 34,139,34
32	chr22 17742142 17752656 14388_4970 0 . 17742142 17752656 34,139,34
33	chr22 18166460 18172712 14327_4410 0 . 18166460 18172712 34,139,34
	**LCR regions**
	browser position chr22:16309492-21775472
	track name="LCR" description="Low Copy Number Repeat Sequences" visibility=2 itemRgb="On"
1	chr22 17276972 18600656 LCR-A_LCR-B 0 . 17276972 18600656 0,0,0
2	chr22 17276972 19712953 Classic_Deletion 0 . 17276972 19712953 0,0,0

Table S3.

Table S3A: 4q22.1deletions (<10MB) coordinates for UCSC genome browser custom tracks (genome build GRCh37/hg19, February 2009)

Genome coordinates for Figure 3A/B are listed in this Table S3A/B.
Cases with neurodevelopmental delay, ASD, and/or other morphological phenotypes:

No.	Type	Size (bp)	Chrom.	Start	Stop	ID	Phenotype	Reference
1	Deletion	67,193	chr4	90791345	90858538	3521_3	N/A	[3]
2	Duplication	177,945	chr4	90527679	90705624	CV251304	Abnormality of the face, Intellectual disability	DECIPHER
3	Deletion	754,432	chr4	90458652	91213084	VCV000146171	Developmental delay AND/OR other significant developmental or morphological phenotypes	ClinVar
4	Deletion	754,432	chr4	90458652	91213084	nsv529189	Developmental delay AND/OR other significant developmental or morphological phenotypes	[5]
5	Deletion	884,797	chr4	90272120	91156917	nsv949454	NA	[6]
6	Deletion	996,780	chr4	90168566	91165346	nsv1323206	NA	[7]
7	Deletion	999,006	chr4	90167781	91166787	nsv1012406	NA	[8]
8	Duplication	4,065,651	chr4	88265504	92331155	CV342092	Duane anomaly, Growth delay, Hemivertebrae, Horseshoe kidney, Vertebral segmentation defect	DECIPHER
9	Deletion	5,756,752	chr4	89571443	95328195	CV337659	Global developmental delay, Joint hypermobility, pes planus	DECIPHER
10	Deletion	6,251,589	chr4	88295927	94547516	CV339430	Agenesis of corpus callosum, porencephalic cyst	DECIPHER
11	Deletion	6,966,581	chr4	90005204	96971785	VCV000562945	N/A	ClinVar
12	Deletion	7,231,379	chr4	85839771	93071150	VCV000396146	Developmental delay AND/OR other significant developmental or morphological phenotypes	ClinVar
13	Deletion	8,344,282	chr4	89891197	98235479	VCV000443904	Oculomotor apraxia Impaired social interactions	ClinVar

							Delayed speech and language development<br>Delayed gross motor development	
14	Deletion	8,523,827	chr4	86370518	94894345	VCV000152923	Developmental delay AND/OR other significant developmental or morphological phenotypes	ClinVar
15	Deletion	9,004,389	chr4	82210925	91215314	CV994_2	Broad palm, Intellectual disability, Narrow nasal bridge, Short foot, Short palm, Short philtrum, Short stature, Tapered finger, Thin lower lip vermilion	DECIPHER

DECIPHER: https://decipher.sanger.ac.uk/
ClinVar: https://www.ncbi.nlm.nih.gov/clinvar/

Count	Genome Browser Custom Tracks (GRCh37/hg19)
	browser position chr4:81985657-98524436
	track name="SNCA - 4q22.1" description="SNCA CNV cases" visibility=2 itemRgb="On"
1	chr4 90791345 90858538 3521_3 0 . 90791345 90858538 65,105,225
2	chr4 90527679 90705624 CV251304 0 . 90527679 90705624 65,105,225
3	chr4 90458652 91213084 VCV000146171 0 . 90458652 91213084 34,139,34
4	chr4 90458652 91213084 nsv529189 0 . 90458652 91213084 34,139,34
5	chr4 90272120 91156917 nsv949454 0 . 90272120 91156917 34,139,34
6	chr4 90168566 91165346 nsv1323206 0 . 90168566 91165346 34,139,34
7	chr4 90167781 91166787 nsv1012406 0 . 90167781 91166787 34,139,34
8	chr4 88265504 92331155 CV342092 0 . 88265504 92331155 65,105,225
9	chr4 89571443 95328195 CV337659 0 . 89571443 95328195 34,139,34
10	chr4 88295927 94547516 CV339430 0 . 88295927 94547516 34,139,34
11	chr4 90005204 96971785 VCV000562945 0 . 90005204 96971785 34,139,34
12	chr4 85839771 93071150 VCV000396146 0 . 85839771 93071150 34,139,34
13	chr4 89891197 98235479 VCV000443904 0 . 89891197 98235479 34,139,34
14	chr4 86370518 94894345 VCV000152923 0 . 86370518 94894345 34,139,34
15	chr4 82210925 91215314 CV994_2 0 . 82210925 91215314 65,105,225

Table S3B: Small SNCA deletions/duplication coordinates for UCSC genome browser custom tracks (genome build GRCh37/hg19, February 2009)

Genome Browser Custom Tracks (GRCh37/hg19)	Reference
Duplications	
browser position chr4:88169442-92453245	
track name=Duplications="Duplications" itemRgb=On	
chr4 90791345 90858538 3521_3 0 . 90791345 90858538 65,105,225	[3]
chr4 90527679 90705624 CV251304 0 . 90527679 90705624 65,105,225	DECIPHER
SNPs/SSV	
browser position chr4:88169442-92453245	
track name=SNPs description="SNPs/SSVs" itemRgb=On	
chr4 90678541 90678541 rs2736990 0 . 90678541 90678541 0,0,0	[9]
chr4 90646886 90646886 rs356165 0 . 90646886 90646886 0,0,0	[9]
chr4 90637601 90637601 rs356219 0 . 90637601 90637601 0,0,0	[9]
chr4 90757394 90757394 rs3756063 0 . 90757394 90757394 0,0,0	[9]
chr4 90674431 90674431 rs356168 0 . 90674431 90674431 0,0,0	[9]
chr4 90639515 90639515 rs11931074 0 . 90639515 90639515 0,0,0	[9]
chr4 90647278 90647278 rs17016074 0 . 90647278 90647278 0,0,0	[9]
chr4 90767039 90767305 Rep1-allele 0 . 90767039 90767305 0,0,0	[9]
ECR	
browser position chr4:88169442-92453245	
track name="ECR Regions" description="Evolutionarily Conserved Regions"	
chr4 90614642 90614787 D1	[10]
chr4 90614642 90614787 D2	[10]
chr4 90629790 90630480 D3	[10]
chr4 90636848 90637316 D6	[10]
chr4 90659197 90659350 I2	[10]
chr4 90674661 90675121 I5	[10]
chr4 90675762 90675891 I6	[10]
chr4 90682267 90682378 I8	[10]
chr4 90721509 90721763 I12	[10]
chr4 90785647 90785975 U3	[10]
chr4 90789074 90789786 U4-1	[10]
chr4 90791038 90791735 U4-3	[10]

Table S3C: SNCA deletions/duplication coordinates for UCSC genome browser custom tracks (genome build GRCh37/hg19, February 2009)

Genome Browser Custom Tracks (GRCh37/hg19)
Triplication
browser position chr4:85000000-98000000
track name="Iowa" description="SNCA Triplication" visibility=2 itemRgb="On"<br>chr4 89337388 91047146 20163_1672002 0 . 89337388 91047146 255,0,0
track name="Lister" description="SNCA Triplication" visibility=2 itemRgb="On"<br>chr4 90302002 91143727 20163_1672002 0 . 90302002 91143727 255,0,0
Duplication
track name="ND40065" description="SNCA Duplication" visibility=2 itemRgb="On"<br>chr4 86743290 94654224 20163_1672002 0 . 86743290 94654224 0,0,255

Table S4. Molecular, morphological, functional, and behavioral phenotypes in murine alpha-synuclein knockout models

		**α-syn KO**	**Double KO (α-β) or (α-γ)**	**Triple KO (α-β-γ)**
Brain architecture and neuronal morphology	Developing brain	Not reported	Not reported	Not reported
	Young/adult (1-14 months)	Normal [11]	Normal in α-β double KO [12]	Normal [13]
	Old (≥24 months)	Not reported	Not reported	Normal [13]
Synaptic topography	Developing brain	Not reported	Not reported	Not reported
	Young/adult (1 - 14 months)	No gross alteration on synaptic terminals, synaptic vesicles in striatum unchanged [11]	Vesicle number and size remained unchanged [12], no significant changes in presynaptic bouton area, density of vesicles (α-β double KO) [12]	30% decreased synaptic termini [13], 28% decrease in presynaptic terminal area [13], unaltered synaptic density [13]
	Old (≥24 months)	Not reported	Not reported	Unaltered synaptic density [13]
Electrophysiology	Developing brain	Not reported	Not reported	Not reported
	Young/adult (1 - 14 months)	Not reported	Not reported	Decreased conduction velocity, excitability [13]
	Old (≥24 months)	Not reported	Not reported	Not reported
Behavior	Young/adult (1 - 14 months)	Reduced learning ability, disturbance of spatial memory [14], anxiety behavior [15], no altered locomotor activity in novel environment [11]	Decrease motor performance (α-β double KO) [16]	Hyperactive in novel environments [17], decrease motor performance [16]
	Old(≥ 24 months)	Motor coordination not impaired [18]	Not reported	Not reported
	Developing brain	Reduction on the dopaminergic neurons (33%) at embryonic day 13.5 [19]	Not reported	Not reported

Midbrain dopaminergic neurons; count and morphology	Young/adult (1 - 14 months)	No difference [11]	No difference in α-β double KO [12], decrease in TH+ neurons in SNpc in α-γ KO [20]	No difference [17], no change on TH+ neurons in the SNpc [16]
	Old (≥24 months)	Decreased of TH+ fibers in the dorsal striatum [18], decrease number of neurons [16]	Reduction of TH+ neurons in the SNpc (α-β double KO) [16]	Mild decrement [13], no change on TH+ neurons in SNpc [16]
Striatal dopamine	Developing brain	Not reported	Not reported	Not reported
	Young/adult (1 - 14 months)	Reduction of striatal dopamine (18%) [11,14], attenuation of dopamine dependent locomotor response to amphetamine [11]	Not reported	Not reported
	Old (≥24 months)	Reduction of 36.2% (24 mo)[18], around 20% decrement [16]	Reduction of 18% [12], around 25% reduction [16] (α-β double KO)	Around 30% decrease [16]
Survival and gross	Developing brain	Not reported	Not reported	Not reported
	Young/adult (1 - 14 months)	Viable, fertile, and normal in size [11]	No changes on survival in α-β double KO [12]	10% decrease survival rate [13], increased mortality (12%) by 12 months [13], unaltered overall survival rate [16]
	Old (≥24 months)	Not reported	Not reported	Unaltered survival rate [16]
β-and or γ-syn	Developing brain	Not reported	Not reported	Not reported
	Young/adult (1 - 14 months)	No changes on gross or subcellular distribution [11], increment on β-syn in midbrain [20]	50% increase of γ synuclein in α-β double KO [12], increase of β synuclein in α-γ KO mice [20]	Not reported
	Old (≥24 months)	Not reported	Not reported	Not reported
	Developing brain	Not reported	Not reported	Not reported

Synaptic proteins	Young/adult (1 - 14 months)	Not reported	30% increase in 14-3-3e protein and complexines in α-β double KO [12]	Increase in complexin II, synapsin IIb, and 14-3-3 β and ε isoforms [13]
	Old (≥24 months)	Rab3a, synaptophysin, syn1 were unaltered [11]	Not reported	Decrease of complexin II, synapsin IIb [13], 14-3-3 β and ε isoforms, changes in SNARE proteins (SNAP-25) and synaptobrevin-2 [13], decrease in synaptobrevin-2 [21], increase in CSPα [21], decrease in SNARE complex [21]
Other dopamine metabolites and transporters	Developing brain	Not reported	Not reported	Not reported
	Young/adult (1 - 14 months)	Not reported	DOPAC and HVA levels unchanged, unchanged levels of 5-HT [12], (in α-β double KO or α-γ KO) [20]	Not reported
	Old (≥24 months)	Unchanged, downregulation of DAT [18]	Not reported	Unchanged [16]
MPTP toxicity resistance	Young/adult (1 - 14 months)	Resistance to MPTP toxicity [20]	Resistance to MPTP toxicity in (α-β double KO) [20]	Not reported
	Old (≥24 months)	Not reported	Not reported	Not reported

References

1. Zafar, F.; Valappil, R.A.; Kim, S.; Johansen, K.K.; Chang, A.L.S.; Tetrud, J.W.; Eis, P.S.; Hatchwell, E.; Langston, J.W.; Dickson, D.W., et al. Genetic fine-mapping of the Iowan SNCA gene triplication in a patient with Parkinson's disease. *NPJ Parkinsons Dis* **2018**, *4*, 18, doi:10.1038/s41531-018-0054-4.
2. Schaaf, C.P.; Sabo, A.; Sakai, Y.; Crosby, J.; Muzny, D.; Hawes, A.; Lewis, L.; Akbar, H.; Varghese, R.; Boerwinkle, E., et al. Oligogenic heterozygosity in individuals with high-functioning autism spectrum disorders. *Hum Mol Genet* **2011**, *20*, 3366-3375, doi:10.1093/hmg/ddr243.
3. Pinto, D.; Delaby, E.; Merico, D.; Barbosa, M.; Merikangas, A.; Klei, L.; Thiruvahindrapuram, B.; Xu, X.; Ziman, R.; Wang, Z., et al. Convergence of genes and cellular pathways dysregulated in autism spectrum disorders. *Am J Hum Genet* **2014**, *94*, 677-694, doi:10.1016/j.ajhg.2014.03.018.
4. Clements, C.C.; Wenger, T.L.; Zoltowski, A.R.; Bertollo, J.R.; Miller, J.S.; de Marchena, A.B.; Mitteer, L.M.; Carey, J.C.; Yerys, B.E.; Zackai, E.H., et al. Critical region within 22q11.2 linked to higher rate of autism spectrum disorder. *Mol Autism* **2017**, *8*, 58, doi:10.1186/s13229-017-0171-7.
5. Miller, D.T.; Adam, M.P.; Aradhya, S.; Biesecker, L.G.; Brothman, A.R.; Carter, N.P.; Church, D.M.; Crolla, J.A.; Eichler, E.E.; Epstein, C.J., et al. Consensus statement: chromosomal microarray is a first-tier clinical diagnostic test for individuals with developmental disabilities or congenital anomalies. *Am J Hum Genet* **2010**, *86*, 749-764, doi:10.1016/j.ajhg.2010.04.006.
6. Vulto-van Silfhout, A.T.; Hehir-Kwa, J.Y.; van Bon, B.W.; Schuurs-Hoeijmakers, J.H.; Meader, S.; Hellebrekers, C.J.; Thoonen, I.J.; de Brouwer, A.P.; Brunner, H.G.; Webber, C., et al. Clinical significance of de novo and inherited copy-number variation. *Hum Mutat* **2013**, *34*, 1679-1687, doi:10.1002/humu.22442.
7. Duyzend, M.H.; Nuttle, X.; Coe, B.P.; Baker, C.; Nickerson, D.A.; Bernier, R.; Eichler, E.E. Maternal Modifiers and Parent-of-Origin Bias of the Autism-Associated 16p11.2 CNV. *Am J Hum Genet* **2016**, *98*, 45-57, doi:10.1016/j.ajhg.2015.11.017.
8. Coe, B.P.; Witherspoon, K.; Rosenfeld, J.A.; van Bon, B.W.; Vulto-van Silfhout, A.T.; Bosco, P.; Friend, K.L.; Baker, C.; Buono, S.; Vissers, L.E., et al. Refining analyses of copy number variation identifies specific genes associated with developmental delay. *Nat Genet* **2014**, *46*, 1063-1071, doi:10.1038/ng.3092.
9. Piper, D.A.; Sastre, D.; Schüle, B. Advancing Stem Cell Models of Alpha-Synuclein Gene Regulation in Neurodegenerative Disease. *Front Neurosci* **2018**, *12*, 199, doi:10.3389/fnins.2018.00199.
10. Sterling, L.; Walter, M.; Ting, D.; Schüle, B. Discovery of functional non-coding conserved regions in the alpha-synuclein gene locus. *F1000Res* **2014**, *3*, 259, doi:10.12688/f1000research.3281.2.
11. Abeliovich, A.; Schmitz, Y.; Farinas, I.; Choi-Lundberg, D.; Ho, W.H.; Castillo, P.E.; Shinsky, N.; Verdugo, J.M.; Armanini, M.; Ryan, A., et al. Mice lacking alpha-synuclein display functional deficits in the nigrostriatal dopamine system. *Neuron* **2000**, *25*, 239-252, doi:10.1016/s0896-6273(00)80886-7.
12. Chandra, S.; Fornai, F.; Kwon, H.B.; Yazdani, U.; Atasoy, D.; Liu, X.; Hammer, R.E.; Battaglia, G.; German, D.C.; Castillo, P.E., et al. Double-knockout mice for alpha- and beta-synucleins: effect on synaptic functions. *Proc Natl Acad Sci U S A* **2004**, *101*, 14966-14971, doi:10.1073/pnas.0406283101.
13. Greten-Harrison, B.; Polydoro, M.; Morimoto-Tomita, M.; Diao, L.; Williams, A.M.; Nie, E.H.; Makani, S.; Tian, N.; Castillo, P.E.; Buchman, V.L., et al. alphabetagamma-Synuclein triple knockout mice reveal age-dependent neuronal dysfunction. *Proc Natl Acad Sci U S A* **2010**, *107*, 19573-19578, doi:10.1073/pnas.1005005107.
14. Kokhan, V.S.; Afanasyeva, M.A.; Vankin, G.I. alpha-Synuclein knockout mice have cognitive impairments. *Behav Brain Res* **2012**, *231*, 226-230, doi:10.1016/j.bbr.2012.03.026.
15. Cabin, D.E.; Shimazu, K.; Murphy, D.; Cole, N.B.; Gottschalk, W.; McIlwain, K.L.; Orrison, B.; Chen, A.; Ellis, C.E.; Paylor, R., et al. Synaptic vesicle depletion correlates with attenuated synaptic responses to

prolonged repetitive stimulation in mice lacking alpha-synuclein. *The Journal of neuroscience : the official journal of the Society for Neuroscience* **2002**, *22*, 8797-8807.
16. Connor-Robson, N.; Peters, O.M.; Millership, S.; Ninkina, N.; Buchman, V.L. Combinational losses of synucleins reveal their differential requirements for compensating age-dependent alterations in motor behavior and dopamine metabolism. *Neurobiol Aging* **2016**, *46*, 107-112, doi:10.1016/j.neurobiolaging.2016.06.020.
17. Anwar, S.; Peters, O.; Millership, S.; Ninkina, N.; Doig, N.; Connor-Robson, N.; Threlfell, S.; Kooner, G.; Deacon, R.M.; Bannerman, D.M., et al. Functional alterations to the nigrostriatal system in mice lacking all three members of the synuclein family. *The Journal of neuroscience : the official journal of the Society for Neuroscience* **2011**, *31*, 7264-7274, doi:10.1523/JNEUROSCI.6194-10.2011.
18. Al-Wandi, A.; Ninkina, N.; Millership, S.; Williamson, S.J.; Jones, P.A.; Buchman, V.L. Absence of alpha-synuclein affects dopamine metabolism and synaptic markers in the striatum of aging mice. *Neurobiol Aging* **2010**, *31*, 796-804, doi:10.1016/j.neurobiolaging.2008.11.001.
19. Garcia-Reitboeck, P.; Anichtchik, O.; Dalley, J.W.; Ninkina, N.; Tofaris, G.K.; Buchman, V.L.; Spillantini, M.G. Endogenous alpha-synuclein influences the number of dopaminergic neurons in mouse substantia nigra. *Exp Neurol* **2013**, *248*, 541-545, doi:10.1016/j.expneurol.2013.07.015.
20. Robertson, D.C.; Schmidt, O.; Ninkina, N.; Jones, P.A.; Sharkey, J.; Buchman, V.L. Developmental loss and resistance to MPTP toxicity of dopaminergic neurones in substantia nigra pars compacta of gamma-synuclein, alpha-synuclein and double alpha/gamma-synuclein null mutant mice. *J Neurochem* **2004**, *89*, 1126-1136, doi:10.1111/j.1471-4159.2004.02378.x.
21. Burre, J.; Sharma, M.; Tsetsenis, T.; Buchman, V.; Etherton, M.R.; Sudhof, T.C. Alpha-synuclein promotes SNARE-complex assembly in vivo and in vitro. *Science* **2010**, *329*, 1663-1667, doi:10.1126/science.1195227.
